# Prescription of Physical Activity by General Practitioners in Type 2 Diabetes: Practice and Barriers in French Guiana

**DOI:** 10.3389/fendo.2021.790326

**Published:** 2022-01-10

**Authors:** Stephanie Dranebois, Marie Laure Lalanne-Mistrih, Mathieu Nacher, Liliane Thelusme, Sandra Deungoue, Magalie Demar, Maryvonne Dueymes, Kinan Drak Alsibai, Nadia Sabbah

**Affiliations:** ^1^ Department of General Medicine, Cayenne Hospital Center, Cayenne, French Guiana; ^2^ Department of Nutrition (UTDN-CSO), University Hospital of Guadeloupe, Pointe à Pitre, France; ^3^ Clinical Investigation Center Antilles, Guadeloupe (INSERM CIC 1424), University Hospital of Guadeloupe, Pointe à Pitre, France; ^4^ Clinical Investigation Center Antilles French Guiana (INSERM CIC 1424) Cayenne Hospital Center, Cayenne, French Guiana; ^5^ Department of Endocrinology and Metabolic Diseases, Cayenne Hospital Center, Cayenne, French Guiana; ^6^ EA3593, Amazon Ecosystems and Tropical Diseases, University of Guiana, Cayenne, French Guiana; ^7^ Department of Biology, Immunology and Parasitology, Cayenne Hospital Center, Cayenne, French Guiana; ^8^ Center of Biological Resources (CRB Amazonie), Cayenne Hospital Center, Cayenne, French Guiana; ^9^ Department of Pathology, Cayenne Hospital Center, Cayenne, French Guiana

**Keywords:** type 2 diabetes, prescription, French Guiana, physical activities, general practitioner, practice

## Abstract

**Background:**

General practitioners (GPs) are the major primary healthcare players in the management of type 2 diabetes. In addition to a well-balanced diet, physical activity (PA) appears as a necessary non-medicinal therapy in the management of diabetic patients. However, GPs emphasize several obstacles to its prescription. The aim of this study is to evaluate the practices, barriers, and factors favoring the prescription of PA in type 2 diabetic patients by GPs in French Guiana.

**Method:**

We conducted a cross-sectional descriptive study using a questionnaire, designed to interview 152 French Guiana GPs and describe their practice in prescribing PA in type 2 diabetic patients.

**Results:**

Our results revealed that the prescription of PA as a non-medicinal therapeutic choice in the management of type 2 diabetes was practiced by 74% of the French Guiana GPs. However, only 37% of GPs responded that they implemented the recommendations; indeed, only one-third knew about them. The majority of GPs were interested in PA training, but only 11% were actually trained in this practice. The lack of structure adapted to the practice of PA and the lack of awareness of the benefits of PA in metabolic pathology appeared as the main obstacles to PA prescription.

**Conclusion:**

This study highlights the importance of improving the training of GPs in the prescription of PA, the development of adapted PA structures, and collaboration between the different actors within the framework of the sport-health system in type 2 diabetes in French Guiana.

## Introduction

Type 2 diabetes is a major public health problem in the world, and French Guiana, a French territory in South America, is no exception. The prevalence of pharmacologically treated diabetes mellitus in 2015 in French Guiana was 8%, which is 1.6 times higher than in mainland France (Source SNIRAM-public health 2015). This rate does not take into account diagnosed but untreated diabetic patients and those with no health insurance (1). Although the health insurance system in French Guiana is the same as in mainland France aiming for universal coverage, intense immigration leaves a fraction of persons uninsured even though they may benefit from government insurance after 3 months and even before for urgent health matters (2). Diabetes-related morbidity is 1.9 times higher in French Guiana than in mainland France (3). The cultural westernization increases sedentary lifestyles, physical inactivity, and associated chronic diseases such as diabetes and obesity (4). Moreover, excess weight and obesity are recognized as major risk factors for the development of type 2 diabetes, with prevalence curves increasing in parallel with weight. In French Guiana, the prevalence of obesity was 18% in 2014 compared to 12% in mainland France.

According to the 2009 World Health Organization (WHO) report, 27% of type 2 diabetes is attributable to physical inactivity, even though the beneficial effects and impacts of physical activity (PA) in type 2 diabetic patients are widely recognized (5, 6). Since 2011, the French National Health Authority has promoted PA by including it as a non-medicinal therapy and as an integral part of the care pathway for type 2 diabetes, to help control risk factors and prevent metabolic pathologies. The 2016 French law of “Health System Modernization” states that general practitioners (GPs) may prescribe adapted physical activity (APA) as part of the care pathway for patients with long-term disease (LTD). The paramedical health professionals—masseur-physiotherapists, occupational therapists, and psychomotor therapists—as well as health professionals with a diploma in the field of APA called Adapted Physical Activity Teachers (APATs), are also authorized to prescribe APA. The role of the intervener is to enable the patient to adopt a regular physically active lifestyle safely, progressively, and in a personalized manner, in order to reduce the risk factors and functional limitations related to his chronic disease. The goal is to empower the patient in terms of PA practice. The March 3^rd^ 2017 French interministerial instruction defines the areas of competence of the various supervisors, who intervene according to the level of impairment of the patient’s functional capacities. Furthermore, one of the goals of the National Sport-Health Strategy 2019–2024 is to roll out Sport-Health centers throughout the country. However, the Ministry has not defined the relative participation of health insurance, the Ministry of Health and Sports, and other actors.

Despite these measures, the prescription of PA and its practice is still scarcely promoted. The French Overseas Health barometer study in 2014 revealed that only 36% of French Guianese persons practiced regular physical activity in accordance with recommendations (7). Type 2 diabetes patients, particularly when they are obese, present more obstacles to sustaining an active lifestyle (8). Previous studies highlight some obstacles to the prescription of PA and low patient practice of PA (9, 10). In French Guiana, the low medical density, the low number of specialist physicians, and geographical isolation contribute to the difficulty of accessing healthcare. Furthermore, despite the impetus to develop sport-health centers in mainland France, there is no sport-health center yet in French Guiana. The population is very heterogenous, with half of the population living in poverty, and a mosaic of cultural representations (Creoles, Europeans, Amerindians, Maroons, Haitians, Brazilians, Guyanese, Surinamese, Chinese, and Hmongs) with different practices towards sports reflecting differences in possibility (due to poverty, for example) or cultural emphasis on sports. In French Guiana’s coastal communities, it is mainly private GPs who treat patients with diabetes.

In the epidemiologic context in French Guiana, promoting the prescription and practice of PA is a major objective and challenge for diabetic patients, as well as the complex sociocultural context poverty, multiple cultures, low GP density, but universal health care requires a better knowledge of what the prescribers actually think and do, which may be at odds with the aims of the decree on PA-prescription.

The objective of our study was hence to evaluate the level of prescription of PA in patients with type 2 diabetes in the complex circumstances of French Guiana and to identify barriers for prescription and adherence.

## Materials and Methods

We conducted a cross-sectional descriptive survey of French Guiana GPs between May and August 2019, to describe their practice in PA prescription in patients with type 2 diabetes. The physicians excluded were those who did not practice general medicine. The anonymous survey questionnaire (**Appendix 1**) was administered between May 17 and August 31, 2019, by email or phone, first explaining the project. Two reminders were made for non-respondents.

The drafting of questions was based on the recommendations of the French National Authority for Health, the French Society of Diabetology, and the American Diabetes Association, as well as some previous studies (11).

The 5–10 minute questionnaire was pilot-tested on 10 individuals. The final questionnaire included 36 closed questions, with dichotomous or multiple choices or Likert scales, as well as open responses.

We used the attached weighting for questions with multiple choices; medium of the points obtained per question: no obstacle was 0 points, few obstacles was 1 point, medium obstacles was 2 points, important obstacles was 3 points, and very important obstacles was 4 points.

## Statistical Analysis

The statistical analysis was carried out by using XL Stat software on Excel, and the graphical charts using Microsoft Office software (Word or Excel). Univariate descriptive analysis of data was initially performed, followed by a secondary bivariate analysis using the Chi^2^ independence tests and Fisher’s exact test on contingency tables. The significance level was 5%.

## Results

### Description of the Sample Interviewed

A total of 152 GPs was contacted (104 private GPs, 23 locum GPs, and 25 GPs employed in delocalized health centers) and 81 responded, of which 8 were excluded for not fulfilling the inclusion criteria. The overall response rate was 48%. The profile of responders was the same as that of non-responders. The 73 GPs (42 men and 31 women) were aged 27–73 years with a median of 43 years (43% were under 40 years old; 17% were over 60 years old). The number of patients seen on average per day is described in **Table 1**.

**Table 1:** Characteristics of responding physician.

	GPs (private)	GPs in delocalized health centers	Locum GPs
Number	47 (64.4%)	13 (17.8%)	13 (17.8%)
Mean Age (years)	46 [23–73]	46.2 [23–71]	45.8 [24–69]
<40 years old	14 (29.8%)	9 (69.2%)	9 (69.2%)
≥60 years old	12 (25.5%)	3 (23.1%)	2 (15.4%)
Sex			
Men	28 (59.6%)	7 (53.8%)	7 (53.8%)
Women	19 (40.4%)	6 (46.2%)	6 (46.2%)
Number of patients/day			
More than 30	22 (46.8%)	2 (15.4%)	2 (15.4%)
Between 20 and 30	16 (34%)	8 (61.5%)	9 (69.2%)
Between 10 and 20	8 (17%)	3 (23.1%)	2 (15.4%)
Less than 10	1 (2.1%)	0 (0.0%)	0 (0.0%)
GPs Physical activity			
PA practice	38 (80.9%)	12 (92.3%)	8 (61.5%)
PA regular	21 (44.7%)	7 (53.8%)	8 (61.5%)
Medical degrees			
Sport medicine	3 (6.4%)	0 (0.0%)	1 (7.7%)
University degree of diabetology	8 (17%)	2 (15.4%)	1 (7.7%)

GP, general practitioner.

Fifty-eight (79%) said they practiced PA themselves, and 36 (49.3%) claimed that they reached WHO’s objectives regarding regular PA; 4 (6%) had a sports medicine diploma and 11 (15%) a diploma in diabetology (**Table 1**).

### Description of the GPs Practice

All GPs declared recommending PA, and 98.6% expected real benefits from PA as a non-medicinal therapy. Concerning the recommendations, 36% were aware of the existence of the “French National Authority for Health Guide 2018” for the prescription of PA and sport for adults, 34% of the existence of the “French National Authority for Health Guide 2018” on the prescription of PA and sport in patients diagnosed with type 2 diabetes, and 26% are aware of decree n° 2016-1990 dated December 30, 2016, on the “dispensing conditions of adapted physical activity prescribed by general practitioners for patients with a long-term condition.”

More than a third (37%) of GPs said they applied the content of these recommendations. Two-thirds (66.6%) of GPs who applied the recommendations felt comfortable *versus* 34.7% of those who did not apply the recommendations (p=0.008). Overall, 76.6% of private GPs prescribed PA *versus* 46% of employed GPs (p=0.028), and 71% of them (private GPs) said they had already prescribed it for patients diagnosed with type 2 diabetes. However, “prescription” was predominantly oral advice (95%), giving contacts of a sport-health professional (23%), and the handing out of documents (18%). Written prescriptions only represented 13% of cases.

Being convinced that their recommendations had an impact was significantly associated with the use of PA prescription as a non-medicinal therapeutic choice (p=0.002): 81% of GPs who were convinced of the impact of PA recommendation used PA as a non-medicinal therapeutic choice against 30% of those who did not believe in this impact (**Table 2**). The practice of regular PA by the physician was associated with the view that his recommendations had an impact (p=0.001). All physicians (100%) who practiced regular PA thought their recommendations impacted patient health *versus* 70% of physicians who did not practice regular PA.

**Table 2:** Impact of GPs’ belief.

		Number of GPs (%) convinced that the recommendations have an impact	
PA prescription	*No n=10*	3 (30.0%)	*p=0.005*
	*Yes n=63*	49 (77.8%)	
PA as non-medicinal therapy	*No n=10*	3 (30.0%)	*p=0.002*
	*Yes n=63*	51 (81.0%)	
GP**’**s practice of regular PA^*^	*No n=37*	27 (73.0%)	*p=0.001*
	*Yes n=36*	36 (100%)	

PA, physical activity.

*GP’s practice of regular PA: three times a week, minimum 45 min per session.

With regard to training on medical prescriptions: 46% of GPs thought they felt at ease prescribing PA, whilst only 11% had already had training on PA prescription. However, 90% of GPs wished to have specific training, 97% of GPs thought general practitioners should be the foremost professionals for the management of type 2 diabetes, and 90% approved the introduction of a sport-health module in general medicine studies.

The time spent per prescription for physical activity is described in **Table 3**. The time spent prescribing PA increased with physicians’ age (p=0.006): 47% of physicians over 60 years and older dedicated more time completing the prescription of PA *versus* only 9.4% of physicians under 40 years old. Only three physicians said that they held consultations dedicated to the prescription of PA for type 2 diabetes patients. Two of them had a university degree in diabetology and declared practicing PA themselves, in line with the WHO recommendations.

**Table 3:** Number of GPs according to the time spent prescribing physical activity.

		<2 min^*^	2 to 5 min^*^	Over 5 min^*^	*p*
**Application of recommendations by GPs**	*No*	20 (46.5)	18 (41.9)	5(11.6)	*p<0.0001*
	*Yes*	1(3.8)	12(46.2)	13(50)	

The application of recommendations was associated with the time spent on prescribing (p<0.0001).

**Figure 1:**
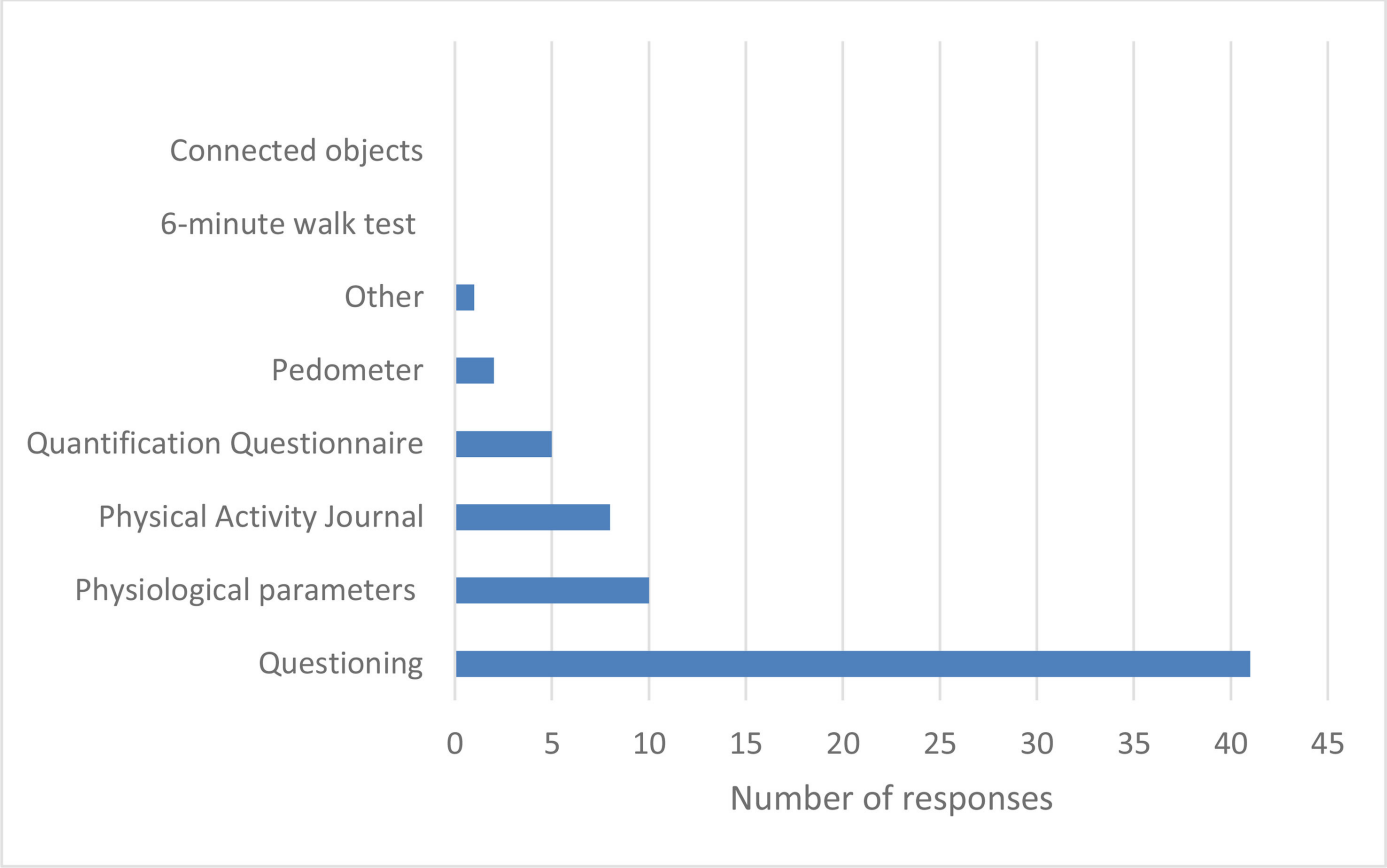
Means of assessing the level of practice and monitoring of PA by GPs. *Minutes per consultation. In brackets are the percentages.

The means used by GPs to assess PA uptake were interviews with patients for 98% of GPs and are described in **Figure 1**. A total of 86.3% of GPs assumed their recommendations benefitted patients, although nearly all GPs believed that over 50% of patients did not follow their recommendations.

#### Free Commentaries

Four GPs added the following items: “give advice to join a club of sports”, “use physiotherapy to reactivate PA after a long period of inactivity”, “sometimes show exercises, and work on patient’s motivation”, and “need to repeat hygiene and nutrition rules during consultation” (**Appendix 2**).

### Limiting Factors in PA Prescription Among GPs

For GPs, the five most important barriers were the lack of adapted structure, the patient’s predictable non-compliance, inadequate supports in French Guiana, lack of support, and lack of training and knowledge (**Table 4**). However, 79.5% of them were not aware of such structures in French Guiana, and 57.5% of the profession of APA teacher, yet 98.6% of GPs were ready to refer them to their patients.

**Table 4:** Limiting factors in PA prescription among GPs.

	Medium*	Median
Lack of structures	2.47	2
Predictable non-compliance	2.08	2
Unsuitable support	1.8	2
Lack of support	1.75	2
Lack of training and knowledge	1.74	2
Lack of time in consultation	1.49	1
Refusal of the patient	1.36	1
No dedicated pricing	1.34	1
Language barrier	1.22	1
Not a reason for a consultation	1.21	1

* Medium of the points obtained per question: no obstacle is 0 point, few obstacles is 1 point, medium obstacles is 2 points, important obstacles is 3 points, and very important obstacles is 4 points.

### Factors Favoring Prescription of PA by GPs

**Figure 2:**
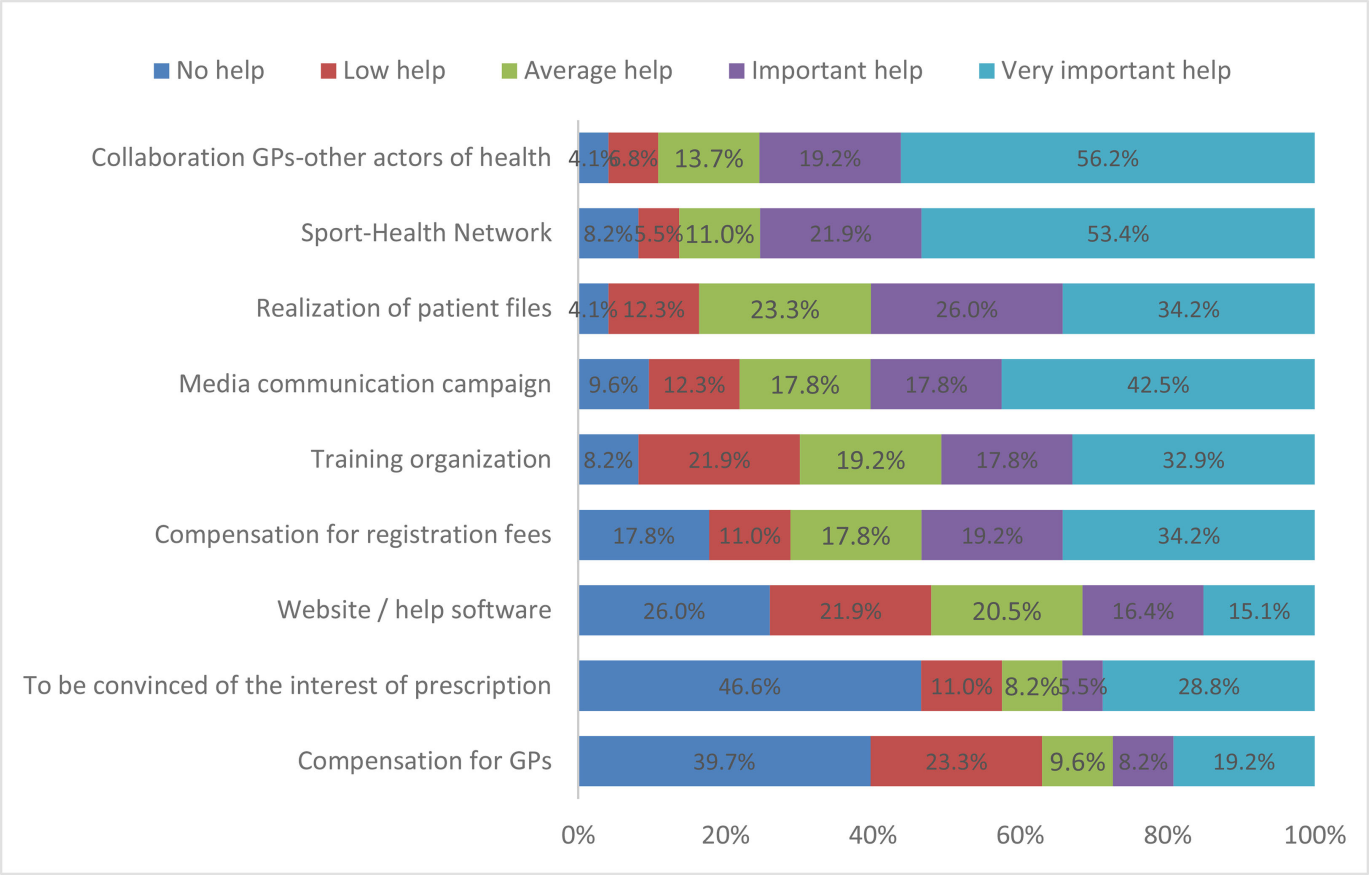
Factors favoring prescription of PA by GPs.

The most useful help for GPs was collaboration between the physician and medical-sport educators, physiotherapists and sports doctors, the sport-health network, the patient information forms, the media communication campaigns, the training of PA prescription, and the reimbursement of PA registration fees (**Figure 2**). We noticed that 84% of GPs thought that compensation of the costs linked to PA prescription would improve patient adherence, and near 55% thought that health insurance reimbursement was a priority (**Figure 2**).

### Causes of Reluctance to Practice PA by Patients, According to GPs

**Figure 3:**
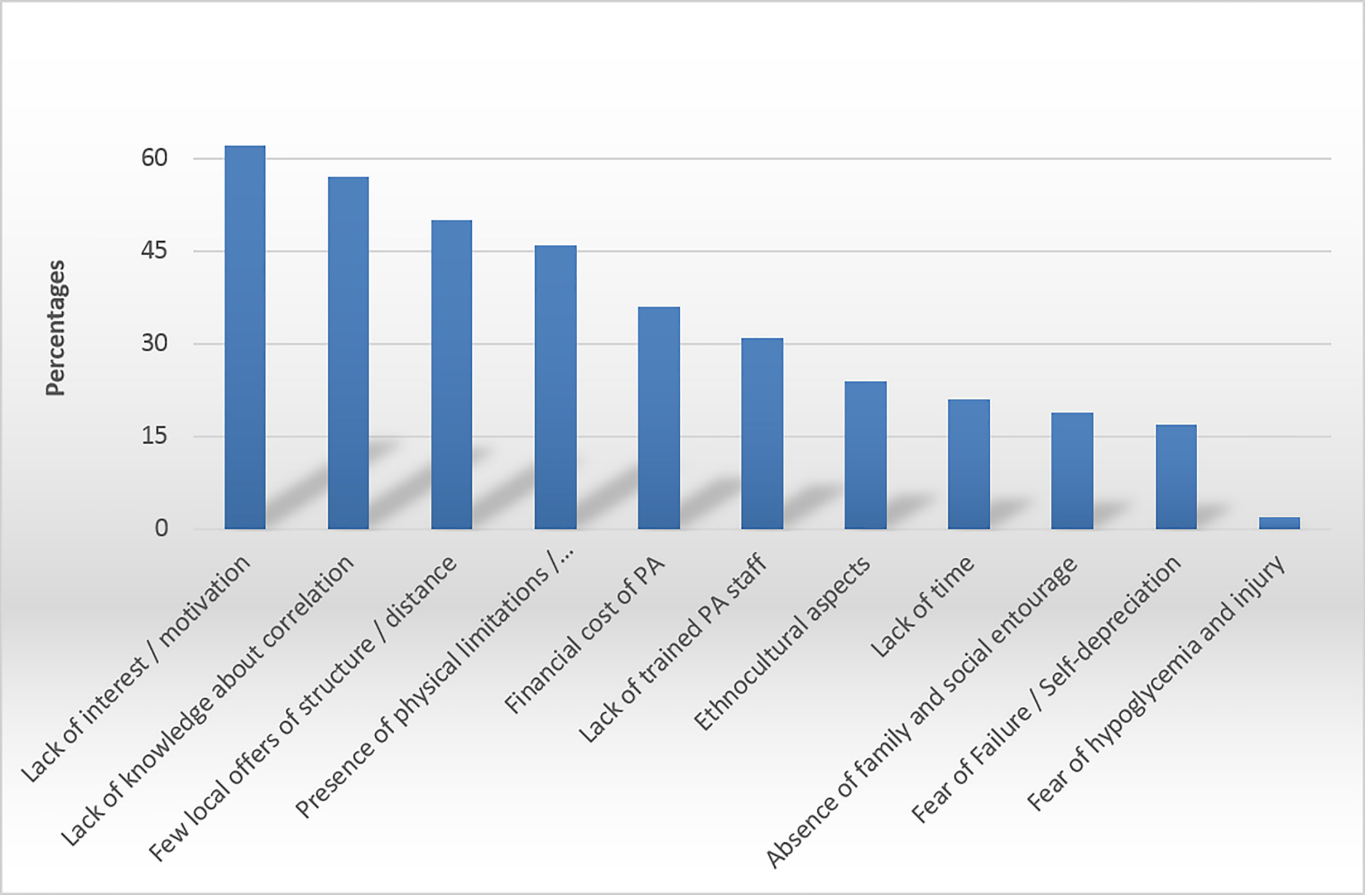
Causes of reluctance to practice PA by patients, according to GPs.

According to GPs, the five main causes of reluctance to practice PA among type 2 diabetes patients in French Guiana were, in descending order: lack of interest/motivation (84.9%), lack of knowledge on the correlation between sport and managing their diabetes (78.1%), few local structures and/or remoteness of these structures (68.5%), presence of physical limitations and comorbidities (63%), and the financial cost of PA (49.3%) (**Figure 3**).

## Discussion

### Description of GPs Practice

The promotion of PA practice among GPs was clearly suboptimal, with two-thirds of GPs unaware of the 2018 guidelines about the prescription of PA and diabetes, and three-quarters unaware of the 2016 French law of “Health System Modernization”. There was no relationship between knowledge of recommendations/decrees and physician-related characteristics. GPs implementing the recommendations felt more competent in the PA prescription than those not following recommendations, and they spent more time doing it. The GP’s low level of training in the fields of sport (5.8%), diabetes (15%), and also in sport-health programs (11%) presumably explains the insufficient awareness of the recommendations.

Very few GPs were in favor of written prescriptions; however, the literature review has shown the effectiveness of this method on increasing practice levels of AP (12). Also, the combination of different means (written prescription, oral advice, and delivery of documents) leads to a significant increase in the practice of PA by patients (13). Our study showed that GPs who follow the recommendations hardly ever spent less than 2 min prescribing; others showed that half of physicians spent less than 5 min prescribing PA (14). On average, a medical consultation lasts 18 min (Drees 2012 data) (15), but there is no exact data concerning the time needed to prescribe PA. The French guidelines (HAS) recommend that a medical consultation for PA should take on average 30 min, but that this can be divided between different consultations (16). Over half of GPs reported assessing the level of PA practice and follow-up for their patients with type 2 diabetes. The preferred means used were questioning in 97.6% of respondents, in agreement with a previous study by Gerin et al. (9).

The use of other means, such as movement counters like pedometers, showed an improvement in patient motivation and PA practice (17). The integration of this simple device may be an eventuality, knowing that according to two previous studies, it seems that it is as well accepted by the patient as by the GPs (18, 19). The collection of the PA level through a questionnaire, diary, or activity notebook also has its advantages. Indeed, its integration as a vital sign in the patient’s medical record in the same way as the other parameters during a consultation is considered as an innovative key action. Several electronic medical software programs have already incorporated this parameter and have found an increase in medical advice, and an impact on weight loss and HBA1C. Given the emergence of connected objects in our societies, new avenues of reflection are being added regarding the use of these means to promote the evaluation and monitoring of PA and its practice (20).

### Representativeness of the Sample and Analysis of Academic Differences

French Guiana has a very low density of doctors, including specialists, despite its size (84,000 km2). Hospital practitioners with a qualification in general medicine sometimes work in specialty services, but mostly in remote health centers that serve isolated villages but are administratively attached to the hospital, where they receive training before going on site.

In our sample, we had 13 doctors who practiced in these remote health centers and were therefore in the academic circuit, and 13 substitute doctors, most of whom had just finished their medical studies and had done their internship in a hospital. These represent one-third of our sample. The medical density in French Guiana is very low (one of the lowest in France), which accentuates the problem of patient follow-up and explains why one of the obstacles to prescribing physical activity and training in it is the lack of time, as doctors are overwhelmed in their daily practice. We have attached a graph with the number of general practitioners, of which there are about 360 (this also includes retired physicians in extended practice who are not working full-time, knowing that some of them are replacements who are not always on the territory, and others are retired and active part-time) (**Appendix 3**), and we were able to analyze 73 GPs.

There are few private practitioners in some of French Guiana’s villages, mainly in the isolated villages of the interior, but also in some coastal towns of French Guiana. In addition to the problem of attractiveness, in some villages there is the problem of an insufficiently large patient base to support a full-time, permanent professional and/or permanent professional; furthermore, the lack of health insurance coverage by much of the population in remote villages and the frequent precariousness do not facilitate the installation of private practices. In eight of these communes (Camopi, Papaïchton, Ouanary, Saint-Elie, Saül, Iracoubo, Awala-Yalimapo, and Grand Santi), there is no health professional in town (Map of French Guiana in **Appendix 4**).

### Factors Favoring Prescription of PA by GPs

Although being aware recommendations were associated with advising and prescribing PA in a study in the USA (21), others argued that despite being informed of the recommendations for good practice in type 2 diabetes, GPs do not adhere to them (22). The present study pointed out that the recommendations barely addressed the subject of PA, which may explain the gap between accepted benchmarks and physician practice. The majority of GPs assumed their recommendations had an impact on their patients, but almost all also believed that over 50% of their patients did not follow their prescriptions. It has been demonstrated that physician advice is associated with increased patient compliance (23). PA prescription is logically associated with dietary advice (24). GPs doubt the effectiveness of their recommendations given the barriers they encounter (25).

Being convinced of the value of PA was not found to be a strong factor in favor of PA prescription, yet this factor has an influence on patient prescribing and practice (**Table 2**). It has been shown that the more barriers the GPs have to prescribe PA, the more barriers the patient will have to practice it, thus resulting in lower levels of PA (14). Although the value of PA no longer needs to be demonstrated in terms of primary, secondary, and tertiary prevention (24, 26–29), some GPs express skepticism about its usefulness (30). Being convinced of the value of PA and its practice among physicians is associated with a better perception of the impact of PA recommendations on their patients (31).

Although some hospital programs exist in French Guiana, there is no regional sport-health center for patients followed by private practitioners. As far as the teaching of PA is concerned, over half of GPs (57.5%) were not aware of the profession of APAT, but nearly all would be ready to refer their patients to them. The collaboration between health professionals was the first factor favoring the prescription of PA voiced by the GPs in our study. Recognition of the APAT is already well established and integrated in health systems in Europe, the United States of America (USA), and Australia. However, in other countries, like China, APAT is not yet recognized by the state (32). In France, the APAT is an accredited professional, trained in APA, and currently plays a central role in PA prescription program. The evaluation of different methods has demonstrated the effectiveness of programs in terms of patient adherence and the change towards an active lifestyle, with an increase in their level of experience. A review of the literature by Bullard et al. showed a 77% average rate of adherence to programs (33). In 2018, the French Society of Professionals APA created an online directory identifying private APATs; however, no APAT in French Guiana is listed.

### Limiting Factors in PA Prescription Among GPs

Our results concerning the obstacles to the prescription of PA, and patient adherence are very similar to the literature by Gérin *et al*. (9) and Duclos *et al*. (10), except on one notable point, which is the “lack of time” item. In fact, the time item was not found to be one of the main obstacles to the prescription of PA in our study, whereas it was in other studies (9, 11); one of the main obstacles to the prescription of PA was the lack of teaching materials and resources.

It is assumed that even if GPs in French Guiana had more time, the PA prescription would not be more important when we take into consideration all the other obstacles highlighted, especially the lack of networks and structures that are most important in our region. According to practically all GPs in the study, it appears important to have adapted structures for referring type 2 diabetic patients for PA. The lack of structure was the first obstacle to the prescription of PA by GPs, but also an obstacle to the adherence of patients to PA for 68.5% of GPs. The development of sport-health networks was the second measure requested by GPs to help prescription. The sport-health networks and structures provide orientation adapted to the patient, in appropriate places, and thus facilitate the maintaining of and commitment to practicing PA. For GPs, these structures enable other healthcare professionals to work together. Indeed, in our study, half of GPs felt alone when prescribing PA, and the majority of GPs were not aware of structures towards which they could refer their patients.

Since the 2016 decree, no dedicated pricing system has been planned for prescribing GPs. Yet the GPs in our study did not consider it a constraint or an incentive. While some suggest such prescription did not require pricing, others viewed compensation of GPs as an incentive to prescribe PA (34). The Ministry of Health envisages a reform of the French healthcare system and remunerations with the aim of enhancing the quality of patient care, whilst improving cooperation between health professionals and empowering the patient. It has considered several foreign models based on “disease management” to inspire its reform but has not finalized it (35).

Although almost all GPs believe that GPs are the main professionals in the management of PA on prescription in type 2 diabetes patients, nearly half of physicians think they do not have the appropriate skills to comfortably prescribe PA (31). As elsewhere, a desire for training was hence expressed by the majority of the Guianese physicians. To meet the demands of medical training and with the goal of promoting and facilitating PA on prescription, the French National Authority for Health produced in September 2018 a guide to promote PA on prescriptions (36), as well as a specific frame of reference for PA on prescription in the context of type 2 diabetes.

### Causes of Reluctance to Practice PA by Patients, According to GPs

Some GPs believe that it is not their role to prescribe PA (30). Indeed, in some countries, health professionals other than GPs are authorized to prescribe PA, and others are considering it (37, 38). The first listed obstacle to patient adherence is principally intrinsic factors, mostly lack of interest/motivation. The patient’s foreseeable lack of compliance was found to be an important obstacle to prescription by more than 50% of GPs. This demonstrates the difficulty GPs have with the motivational interview, often because of a lack of training. The second factor is the lack of patient knowledge about the correlation between sport and the management of their diabetes. This element illustrates the need to increase training in TPE in order to improve patients’ knowledge and to increase their adherence to care and treatments. In addition, GPs also identified the presence of physical limitations and comorbidities (2). Additional care given by paramedics and APAT offers a framework of practice supported by trained health professionals, authorized to provide APA to patients with functional limitations, even when these limitations are severe. The absence and/or remoteness of structures was the third factor cited by GPs. According to the 2014 French overseas territories health barometer, 12% of Guianese declared having renounced care because of transportation issues (2). To overcome this obstacle, PA can take place outdoors, and the development of sports facilities in communal areas is important; APA programs at home also seem to be accepted by patients (39). The patient’s cultural resistance by one-third of physicians has also been reported elsewhere and raises the need for culturally appropriate interventions in multicultural French Guiana (40, 41). Finally, the financial cost of PA was mentioned as the fifth obstacle to patient adherence in this study and for 53.4% of physicians, the compensation of patient costs appeared to be a particularly important measure to help prescription.

Half of GPs believed that health insurance should take part in its financing in priority, a greater proportion than elsewhere, presumably because of the major problem of precariousness in French Guiana (3). Hence, 30.9% of Guianese had renounced seeking medical care for financial reasons (2). The socioeconomic context of the patient has been shown to impact the level of patients’ practice (40, 41). This emphasizes the need to overcome the problem of the costs linked to the practice of PA by patients in French Guiana.

Currently, a hybrid model, which combines the different modes of payment, appears to be more suitable to satisfy health professionals, patients, and the state in a common goal of improving the quality of care of chronic diseases.

It would be important to adopt a scheme in French Guiana with a list of approved APAT to whom each GP could refer patients after issuing a prototype prescription, and to set up mobile APAT to fit with territorial constraints. Concerning the limitations of our study, despite reaching a significant proportion of GPs, the sample size was nevertheless small, leading to the imprecision of estimates. Some questions were subject to social desirability bias leading to an overvaluation of positive responses compared with reality. To limit this, responses were anonymous. The method of recruitment by self-administered questionnaire was chosen to reinforce the sense of anonymity, thereby limiting social desirability bias and providing the possibility of responding at the most suitable time for GPs.

A response and formulation bias may appear. The declarative and subjective data and the absence of standardized definitions of some of the terms employed might have biased the estimates. Furthermore, we did not collect the mixed activity of GPs in the questionnaire, whereas analyses between employed and private/self-employed physicians were carried out.

This work emphasizes that we must continue this training effort both within the hospital and especially the doctors of the remote health centers serving isolated populations, especially with experience reviews and clinical cases. However, we also have to work in a sustained way with our private practitioner colleagues who are more isolated than the hospital doctors because there is only one liberal endocrinologist in the whole territory, and although there is a mobile therapeutic education team for the greater Cayenne area, they have difficulties accessing such resources elsewhere.

## Conclusion

Despite its importance in the therapeutic management of type 2 diabetes patients, the prescription of physical activity by general practitioners is insufficient, and several barriers have been highlighted that are often avoidable, and in agreement with the literature. Thus, it seems essential to improve the training of general practitioners in the field of physical activity prescription, to develop therapeutic education in particular by paramedical staff, and to develop cooperation between different healthcare professionals. The establishment of sport-health structures associating caregiver’s assistants and sports educators would be a major asset in the care of type 2 diabetes. Although, health insurance may use financial incentives to develop physical activity, local authorities, in the spirit of health promotion, should provide their citizens sufficient infrastructures to maintain or restore their health through sports, a difficult objective to achieve in a territory where rapid demographic growth and widespread unplanned city expansion create a substantial infrastructure lag.

## Data Availability Statement

The original contributions presented in the study are included in the article/**Supplementary Material**. Further inquiries can be directed to the corresponding author.

## Ethics Statement

Written informed consent was obtained from the individual(s) for the publication of any potentially identifiable images or data included in this article.

## Author Contributions

Conceptualization and design: SDr, NS, ML-M, KD, LT, and MDe. Data collection and curation: SDr and NS. Formal analysis: SDr, NS, ML-M, MN, LT, DS, MDu, and KD contributed to the development of the manuscript: SDr, NS, ML-M, MN, and KD. All authors contributed to the article and approved the submitted version.

## Conflict of Interest

The authors declare that the research was conducted in the absence of any commercial or financial relationships that could be construed as a potential conflict of interest.

## Publisher’s Note

All claims expressed in this article are solely those of the authors and do not necessarily represent those of their affiliated organizations, or those of the publisher, the editors and the reviewers. Any product that may be evaluated in this article, or claim that may be made by its manufacturer, is not guaranteed or endorsed by the publisher.

## Abbreviations

GP, general practitioner; PA, physical activity; APA, adapted physical activity; APAT, adapted physical activity teachers; TPE, therapeutic Patient Education; LTD, long-term disease; CNIL, National Commission for French Data Protection and Liberties.
