# Supplementary material for: Prescription of Physical Activity by General Practitioners in Type 2 Diabetes: Practice and Barriers in French Guiana
Source: Front Endocrinol (Lausanne). 2022 Jan 10;12:790326. doi: 10.3389/fendo.2021.790326 (PMC8784518; doi:10.3389/fendo.2021.790326)
Supplement: Supplementary file 1 [file DataSheet_1.docx]

APPENDIX 1

QUESTIONNAIRE ENGLISH FORM

PRESCRIPTION OF PHYSICAL ACTIVITY IN PATIENTS DIAGNOSED WITH TYPE 2 DIABETES

Dear Colleagues,

As part of my research in diabetology we ask for your participation by answering this questionnaire anonymously.

It will only take you 5-10 minutes.

InFrench Guiana, type 2 diabetes is a real public health problem: the standardized prevalence of diabetes treated pharmacologically in 2015 in Guyana is 9%, i.e. 1.6 times the national rate (Source SNIRAM - Public Health, 2015) .

Although the beneficial effects of regular physical activity have been demonstrated in the management of T2DM (type 2 Diabetes Mellitus), the level of physical activity in diabetic patients remains insufficient.

With the aim of improving the care of these patients, recommandations on physical activity and type 2 diabetes were established by the HAS in September 2018:

https://www.has-sante.fr/portail/upload/docs/application/pdf/2018-10/ref_aps_dt2_vf.pdf.

This study will allow us to establish an inventory concerning the prescription of PA in T2DM patients by general practitioners. We will also seek to determine the obstacles to the practice and prescription of PA, as well as the factors that would favor the prescription.

We thank you in advance for your time and invaluable assistance.

Confraternally,

Stephanie DRANEBOIS,

s.dranebois@gmail.com

Under the direction of Doctor Nadia SABBAH, Endocrinologist at Cayenne Hospital

*o By submitting this form, I agree that the information entered anonymously in this form will be used, processed and exploited to allow the study to be carried out.*

*o In accordance with the Data Protection Act, I am informed that the retention period of my data will not exceed the duration of the study and that I have a right of access, rectification, modification and deletion of data concerning me.*

*QUESTIONNAIRE ENGLISH FORM*

*PRESCRIPTION OF PHYSICAL ACTIVITY IN PATIENTS DIAGNOSED WITH TYPE 2 DIABETES*

*I- KNOWLEDGE AND PRACTICE CONCERNING THE PRESCRIPTION OF PHYSICAL ACTIVITY*

***1- In general, do you recommend physical activity (PA) to your patients ?***

**** Only one answer possible.***

*o Yes*

*o No*

***2- Do you think that PA is an effective non-drug approach: that a real benefit is expected?***

**** Only one answer possible.***

*o Yes*

*o No*

***3- Are you aware of the existence of:***

**** Only one answer possible per line.***

*- HAS Guide 2018 on the promotion, consultation and prescription of physical and sporting activity for health in adults?*

*o Yes*

*o No*

*- HAS 2018 standard on the prescription of physical and sporting activity Type 2 diabetes Mellitus (T2DM)?*

*o Yes*

*o No*

*- Decree n ° 2016-1990 of December 30, 2016 relating to the conditions for dispensing the adapted physical activity prescribed by the attending physician to patients suffering from a long-term illness?*

*o Yes*

*o No*

***4- In general practice, do you apply the content of these recommendations in the context of PA in type 2 diabetic patients ?***

** Only one answer possible.*

*o Yes*

*o No*

***5- Have you already prescribed PA in your type 2 diabetic patients ?***

**** Only one answer possible.***

*o Yes*

*o No*

***6- If yes, what means (s) do you use in your daily practice for this prescription ?***

***(many possible responses)***

*o Written prescription for an adapted physical activity program*

*o Oral advice for an autonomous or supervised AP practice*

*o Indication of contact details for specialized professionals*

*o Distribution of information documents / educational sheets*

*o Other:*

***7- How much time do you spend there during a consultation ?***

** Only one answer possible.*

*o no time spent*

*o less than 2 minutes*

*o 2 to 5 minutes*

*o more than 5 minutes*

***8- Do you sometimes carry out dedicated consultations for PA in these type 2 diabetic patients?***

**** Only one answer possible.***

*o Yes*

*o No*

***9- Do you use PA as a non-drug therapeutic choice in the management of type 2 diabetic patients?***

** Only one answer possible.*

*o Yes*

*o No*

***10- Do you assess the level of practice and follow-up of the PA of your type 2 diabetic patients?***

**** Only one answer possible.***

*o Yes*

*o No*

***11- If yes, which evaluation method (s) do you use?***

***(many possible responses)***

*o Physical activity notebook / diary*

*o 6 minute walk test*

*o Quantification questionnaires*

*o Physiological markers (example: heart rate ..)*

*o Interrogation*

*o Connected objects*

*o Movement counters: Pedometer, Accelerometer*

*o Other:*

***12- Do you think your recommendations have an impact on your type 2 diabetic patients? If so, what is your estimate of the percentage of patients who follow your prescriptions ?***

** Only one answer possible.*

*o Yes, less than 20%*

*o Yes, between 20 and 50%*

*o Yes, more than 50%*

*o No*

***II- OBSTACLES AND PROMOTING FACTORS***

***1- What are obstacles to your prescription of PA?***

*(0: Not a brake, 1: Weak brake, 2: Medium brake, 3: Strong brake, 4: Very strong brake)*

** Only one answer possible per line.*

|  | *0* | *1* | *2* | *3* | *4* |
| --- | --- | --- | --- | --- | --- |
| *Not a reason for consultation?* |  |  |  |  |  |
| *Lack of structures* |  |  |  |  |  |
| *Lack of training / knowledge* |  |  |  |  |  |
| *Patient refusals* |  |  |  |  |  |
| *Materials unsuitable for practice in Guyana* |  |  |  |  |  |
| *No dedicated pricing* |  |  |  |  |  |
| *Lack of supports* |  |  |  |  |  |
| *Foreseeable patient non-compliance* |  |  |  |  |  |
| *Language barrier* |  |  |  |  |  |
| *Lack of time during the consultation* |  |  |  |  |  |

***2- Do you feel that you are alone when it comes to integrating PA in the care of type 2 diabetic patients?***

** Only one answer possible.*

*o Yes*

*o No*

***3- What would help / improve your PA prescription ?***

*(0: No help, 1: Little help, 2: Medium help, 3: Important help, 4: Very important help)*

** Only one answer possible per line.*

|  | *0* | *1* | *2* | *3* | *4* |
| --- | --- | --- | --- | --- | --- |
| *Public media communication campaign* |  |  |  |  |  |
| *Compensation for patient registration fees* |  |  |  |  |  |
| *Organization of training on the prescription of PA* |  |  |  |  |  |
| *Financial compensation for doctors* |  |  |  |  |  |
| *Be convinced of the interest of the prescription of the PA* |  |  |  |  |  |
| *Sport-health network* |  |  |  |  |  |
| *Website / prescribing software* |  |  |  |  |  |
| *Production of explanatory sheets to be given to patients* |  |  |  |  |  |
| *Collaboration between physician and sports medicine educator / physiotherapist / sports medicine service* |  |  |  |  |  |

***4- In your opinion, what are the five main causes of reluctance / obstacles to the practice of PA in type 2 diabetic patients in French Guiana? (Select 5 answers)***

**Many possible responses.*

*o Lack of knowledge on the correlation between sport and the management of their diabetes*

*o Presence of physical limitation and comorbidities*

*o Fear of hypoglycaemia and / or possible risk of injury*

*o Fear of failure or previous failure / Self-deprecation*

*o Financial cost of the PA*

*o Lack of interest / motivation*

*o Ethnocultural aspects*

*o Lack of family and social circle*

*o Few local offers of structure and / or distance from a structure*

*o Lack of trained PA personnel*

*o Lack of time*

***5- Do you think that compensation for the costs associated with prescribing PA on prescription would improve patient adherence? If so, which organization should fund first?***

** Only one answer possible.*

*o Yes, Social security / Health insurance fund*

*o Yes, Mutual / Health insurance*

*o Yes, local authorities*

*o No*

***6- In your opinion, is it important to have a structure to guide type 2 diabetic patients within the framework of PA (example: a sports-health network)?***

** Only one answer possible.*

*o Yes*

*o No*

***7- Do you know of such a structure in French Guiana?***

** Only one answer possible.*

*o Yes*

*o No*

***8- Are you aware of the existence of the therapeutic patient education sector of the Diabetes-Guyana-Obesity network of Cayenne hospital?***

** Only one answer possible.*

*o Yes*

*o No*

***9- Do you know the profession of adapted physical activity teacher (APA) such as medico-sports educators?***

** Only one answer possible.*

*o Yes*

*o No*

***10- Would you be ready to refer a patient to an APA*** *(Adapted physical activity)* ***teacher?***

** Only one answer possible.*

*o Yes*

*o No*

***III- PRESCRIPTION TRAINING***

***1- Do you feel that you have sufficient skills to be comfortable prescribing PA?***

**** Only one answer possible.***

*o Yes*

*o No*

***2- Have you ever had sports-health training on the prescription of PA?***

**** Only one answer possible.***

*o Yes*

*o No*

***3- Would you like to benefit from specific training in this area?***

**** Only one answer possible.***

*o Yes*

*o No*

***4- What type of training would be the most appropriate?***

***(many possible responses)***

*o Evenings or days of seminars*

*o University degree (like University degree of diabetology)*

*o Continuing medical education*

*o MOOC (free online training)*

*o Repository / Guide distributed*

*o Other:*

***5- Do you consider that the general practitioner is a main actor in the management of PA in the T2DM patient ?***

**** Only one answer possible.***

*o Yes*

*o No*

***6- Do you think it would be interesting to include a sport-health module during the training in general medicine?***

**** Only one answer possible.***

*o Yes*

*o No*

***IV- ABOUT YOU***

***1- You are:***

** Only one answer possible.*

*o a woman*

*o a man*

***2- How old are you?***

***3- What is your mode of exercise?***

** Only one answer possible.*

*o Private cabinet*

*o Delocalised health center*

*o Replacement not installed*

***4- How many patients do you see on average per day?***

**** Only one answer possible.***

*o Less than 10*

*o 10 to 20*

*o 20 to 30*

*o More than 30*

***5- In which municipality do you practice ?***

**** Only one answer possible.***

*o All over Guyana*

*o Apatou*

*o Awala-Yalimapo*

*o Camopi*

*o Cayenne*

*o Grand-Santi*

*o Iracoubo*

*o Kourou*

*o Macouria*

*o Mana*

*o Maripasoula*

*o Matoury*

*o Montsinery-Tonnégrande*

*o Rémire-Montjoly*

*o Ouanary*

*o Papaïchton*

*o Regina*

*o Roura*

*o Saint-Elie*

*o Saint-Georges*

*o Saint-Laurent-du-Maroni*

*o Saul*

*o Sinnamary*

***6- Do you practice a physical activity?***

** Only one answer possible.*

*o Yes*

*o No*

***7- Do you think you will achieve the objective of the WHO recommendations for weekly PA?***

***(Recommended regular physical activity: 150 min / week of moderate intensity PA or 75 min / week of high intensity PA)***

**** Only one answer possible.***

*o Yes*

*o No*

***8- Do you have a diploma (University degree,*** ***specialized complementary study diploma...)***

**** Only one answer possible per line.***

*Yes No*

*in sports medicine?*

*in diabetology?*

***9- Free comment:***
