# Supplementary material for: Prescription of Physical Activity by General Practitioners in Type 2 Diabetes: Practice and Barriers in French Guiana
Source: Front Endocrinol (Lausanne). 2022 Jan 10;12:790326. doi: 10.3389/fendo.2021.790326 (PMC8784518; doi:10.3389/fendo.2021.790326)
Supplement: Supplementary file 2 [file DataSheet_2.docx]

APPENDIX 2

FREE COMMENTS

"It is very interesting and indispensable to structure this point in our daily practice as well as possible. Thank you for this very interesting topic! "

"One of the biggest obstacles remains the ridiculous remuneration for consultation which means that a liberal doctor is condemned to slaughter to earn the minimum income he is entitled to expect as long as he remains in the conventional system. With an average consultation time of 15 minutes (often less in Guyana), how do you want to have time to talk about PA to a diabetic who often comes with other problems to solve. "

"Informally, I have diabetic patients who already walk a lot to go shopping because they don't have any means of transportation. For others, however, their advanced age is a limiting factor in getting around.

In my practice, in addition to the physical activity recommended verbally, I ask patients to observe mainly hygienic and dietetic measures. "

"We doctors are isolated, there is a cruel lack of structure and specialist doctors in French Guyana".

" Interest of a dedicated room with equipment (bikes, treadmills etc. + monitors) "

"Physical activity is very important especially for diabetics, but the lack of PA centers, professionals and lack of information is holding people back.

An important point is the lack of liberal doctors (medical desert)".

"It's important to promote this health sport aspect"

"For the indemnity, it's the problem of a global lump sum;

You have to be trained and motivated in physical activity yourself, you have to explain the superiority of diet over drugs;

This work on physical activity can only be coupled with calorie reduction; the passage through a network is most often indispensable, hence the valorization and organization of the network;

However, let's have no illusions, after a while the diabetic is demobilized".

"By being in 100% insurance cover, we relieve the patients of their responsibility regarding diabetes, worsening their life imbalance that they can forget since it has become a disease, therefore not their responsibility but that of the medical body; the prescription of PA achieves the same thing, while it is the basis with dietetics of a more balanced life, therefore putting the patients back in front of their reality, with efforts to be made .... "

"Most of the time the DT2 don't do any sport. I orally prescribe to walk 20 to 30 min 3 / week for already a fitness.

I am not sure that registration in a gym is judicious for patients who have mostly never done any sport (year-round registration).

For women I prescribe zumba or other dance classes.

In my opinion, it is necessary to redo the minimum of activity before considering more intensive sports or those requiring a stronger commitment. "

"I answered no for the sports network because it doesn't reach Grand-Santi, I think that in the isolated communes there should be an offer with activity every day where we can refer patients and others. Setting up associations... Also companions who break the "Western dream" image of the wealth linked to sedentary life.

I don't think that the problem is ethnocultural as it is proposed (at least in my community) because in fact the elderly do physical activities and the young people are largely westernized. Between the two it is a mixed one.

For billing and time is the eternal problem with the therapeutic education... the hospital counts our working time as curative not preventive, we would have to be much more numerous to do both correctly. This can now evolve with the implementation of the public health sector (mobile team) in the CDPS. Something should be offered to them with sports educators trained in the communes who would join the team as employees of the Cayene hospital or the commune.? Go for it!

The general practitioner is the first guarantor of patient follow-up for chronic diseases. All the more so in the isolated communes of French Guyana, the problem is the turnover of doctors in CDPS compared to the coast (private practices) in my opinion. "

"Having discovered during the course of the Diabetology degree in Physical Activity Adapted to diabetics and in particular to those who are overweight, I became aware of what this tool could "bring us" (patients and caregivers) in terms of overall therapeutic efficiency in the management of this pathology, including self-esteem, an essential condition for long-term compliance ... "
