# Supplementary material for: Prescription of Physical Activity by General Practitioners in Type 2 Diabetes: Practice and Barriers in French Guiana
Source: Front Endocrinol (Lausanne). 2022 Jan 10;12:790326. doi: 10.3389/fendo.2021.790326 (PMC8784518; doi:10.3389/fendo.2021.790326)
Supplement: Supplementary file 3 [file DataSheet_3.pdf]

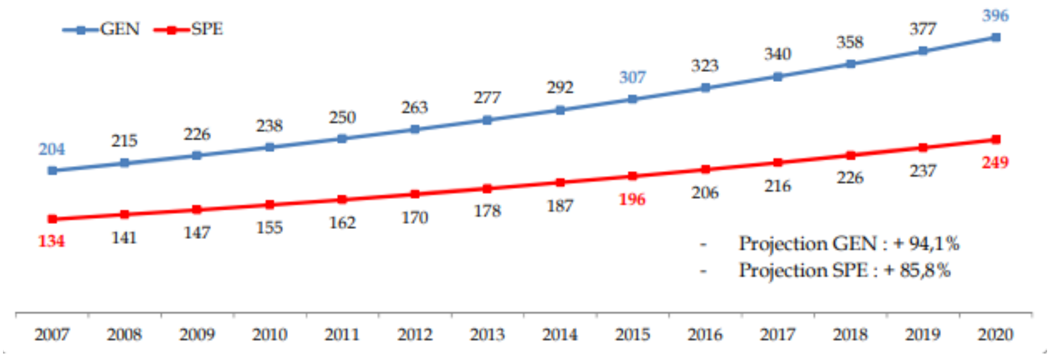

Appendix 3 Number of general practionners and specialist in French Guiana in 2019 and projection

From https://www.conseil-national.medecin.fr/sites/default/files/external-package/analyse_etude/m5d6xw/atlas_dom_com_2015.pdf
