# Supplementary material for: Prescription of Physical Activity by General Practitioners in Type 2 Diabetes: Practice and Barriers in French Guiana
Source: Front Endocrinol (Lausanne). 2022 Jan 10;12:790326. doi: 10.3389/fendo.2021.790326 (PMC8784518; doi:10.3389/fendo.2021.790326)
Supplement: Supplementary file 4 [file DataSheet_4.pdf]

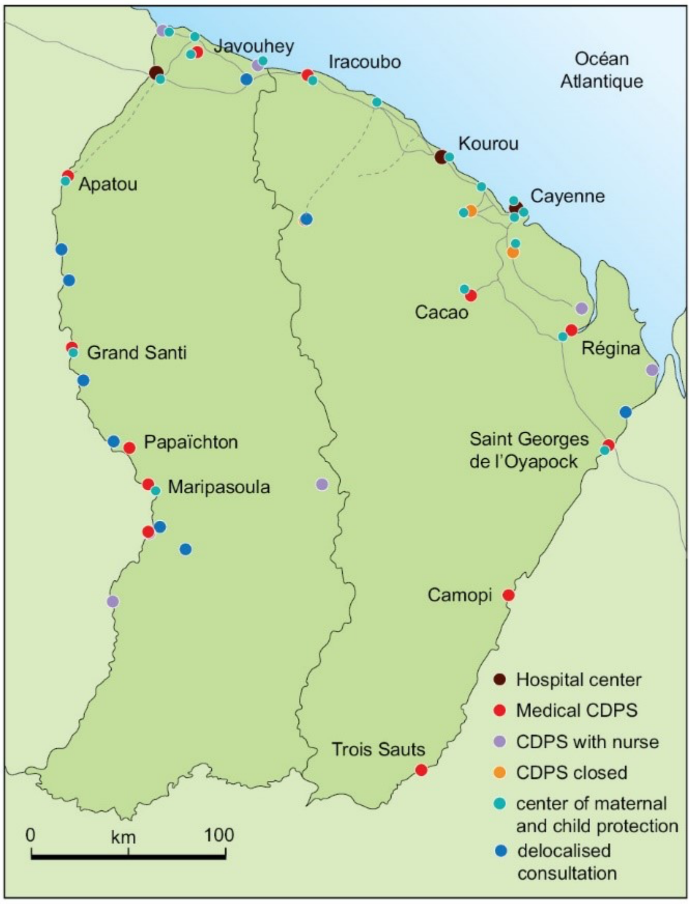

Appendix 4 : Map of French Guiana and the main hospitals and delocalized health centers

CDPS : community health care center

from the accepted publication in progress Frontiers in Endocrinology "Diabetes care in French Guiana: The Gap between national guidelines and reality" Christine Sudre ,Hélène Duplan, John Bukasakakamba Mathieu Nacher, Pascale Peyre-Costa, Nadia Sabbah.
